# Supplementary material for: Elevated mutation rates in multi-azole resistant Aspergillus fumigatus drive rapid evolution of antifungal resistance
Source: Nat Commun. 2024 Dec 16;15:10654. doi: 10.1038/s41467-024-54568-5 (PMC11649685; doi:10.1038/s41467-024-54568-5)
Supplement: Supplementary file 1 — Supplementary Information [file 41467_2024_54568_MOESM1_ESM.pdf]

Supplementary information

Elevated mutation rates in the multi-azole resistant *Aspergillus fumigatus* clade drives rapid evolution of antifungal resistance

Michael J. Bottery, Norman van Rhijn, Harry Chown, Johanna L. Rhodes, Brandi N. Celia-Sanchez, Marin T. Brewer, Michelle Momany, Matthew C. Fisher, Christopher G. Knight, and Michael J. Bromley

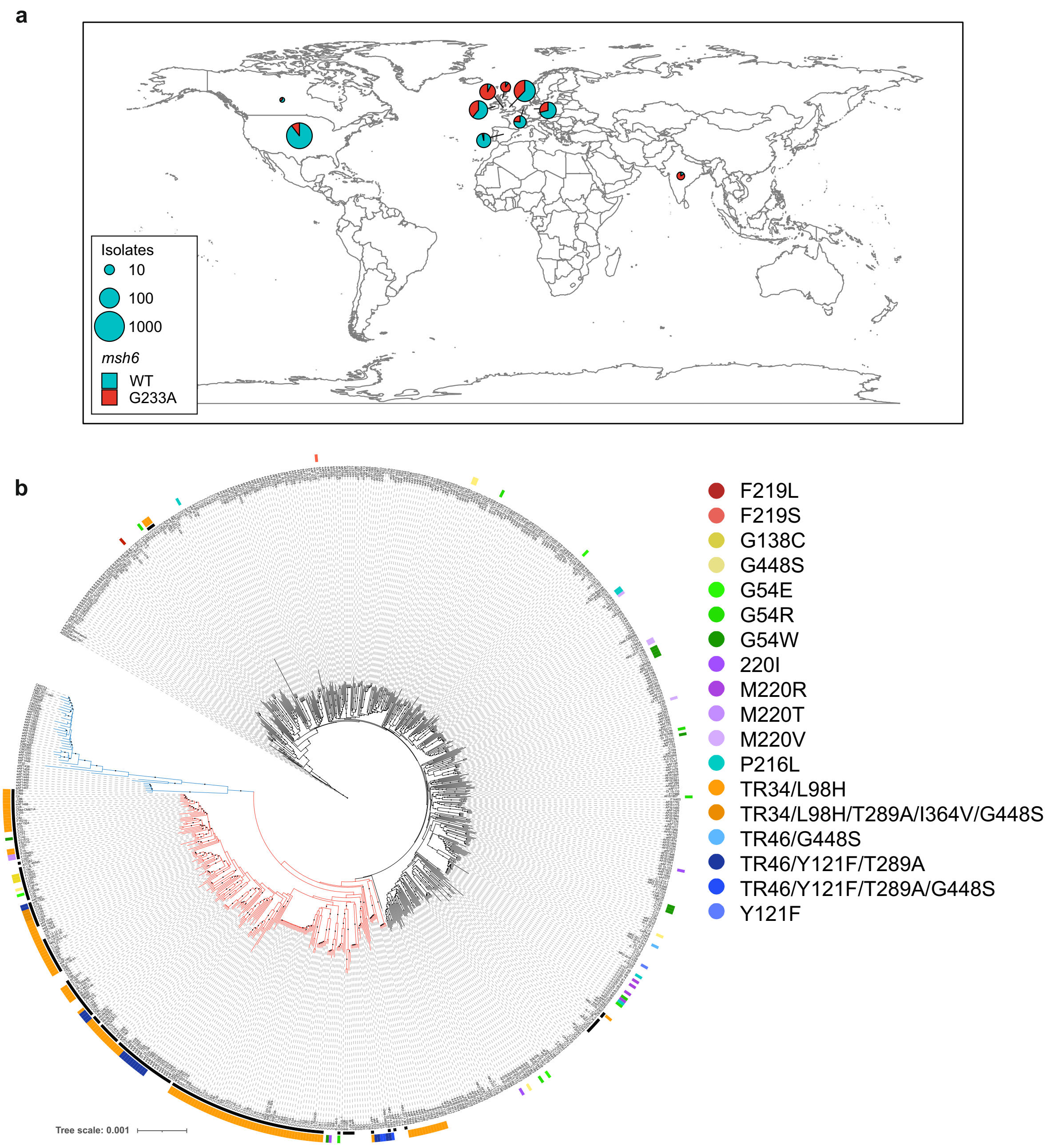

Figure S1 Global presence of *msh6*-G233A within TR_{34}/L98H genotypes. a Map showing the proportion of *msh6* G233A variants by country for 728 publicly available whole genome sequenced *A. fumigatus* isolates show that the mismatch repair variant is observed globally. Size of pie charts scaled by number of isolates. **b** A neighbour joining phylogenetic tree using genome wide SNPs supports the clustering of TR_{34}/L98H genotypes in clade A (red branches) and the association with *msh6*-G233A variant (black points on tip labels) rooted to Af293. Source data are provided as a Source Data file.

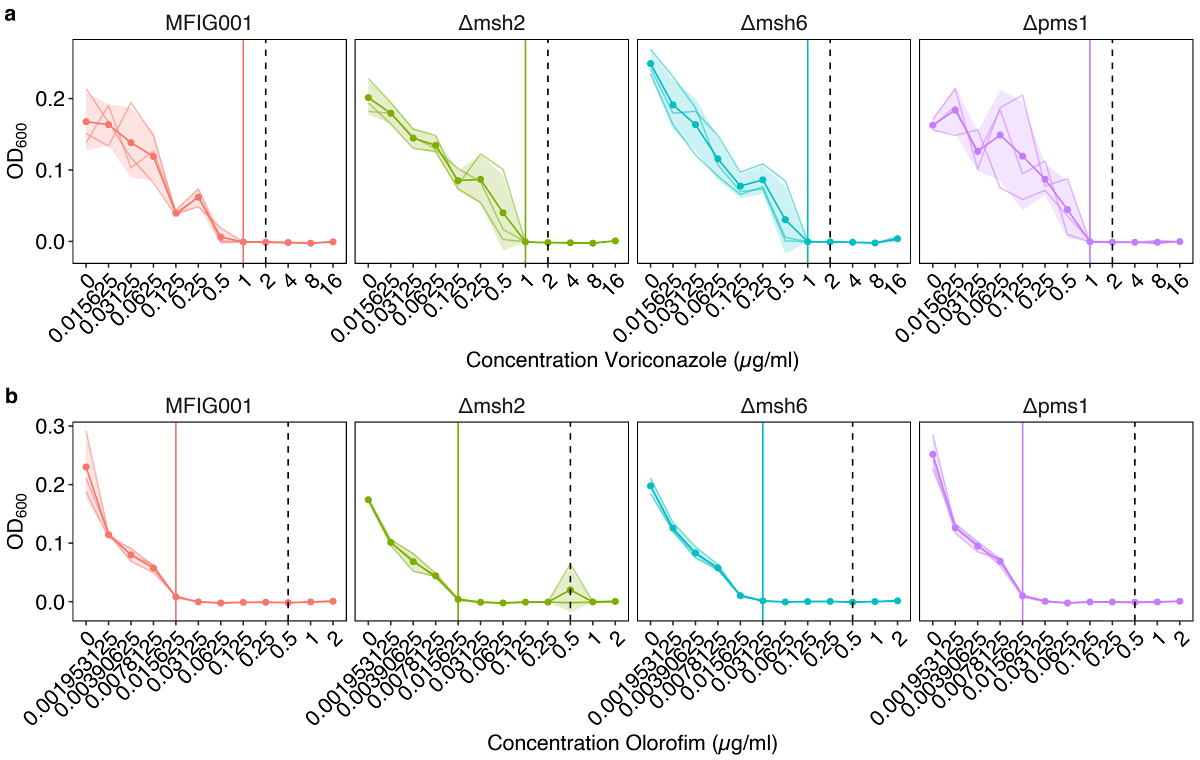

Figure S2 MMR deficient mutants do not show altered MIC to voriconazole or olorofim. a MIC curves of MFIG001 parental strain and *Δmsh2*, *Δmsh6* and *Δpms1* to voriconazole. **b** MIC curves of MFIG001 parental strain and *Δmsh2*, *Δmsh6* and *Δpms1* to olorofim. Bold lines show means of three replicates, each of which are shown separately. Shaded area shows +/- standard deviation. Dashed vertical line shows concentration used to select for resistant mutants, vertical solid line shows MIC. Source data are provided as a Source Data file.

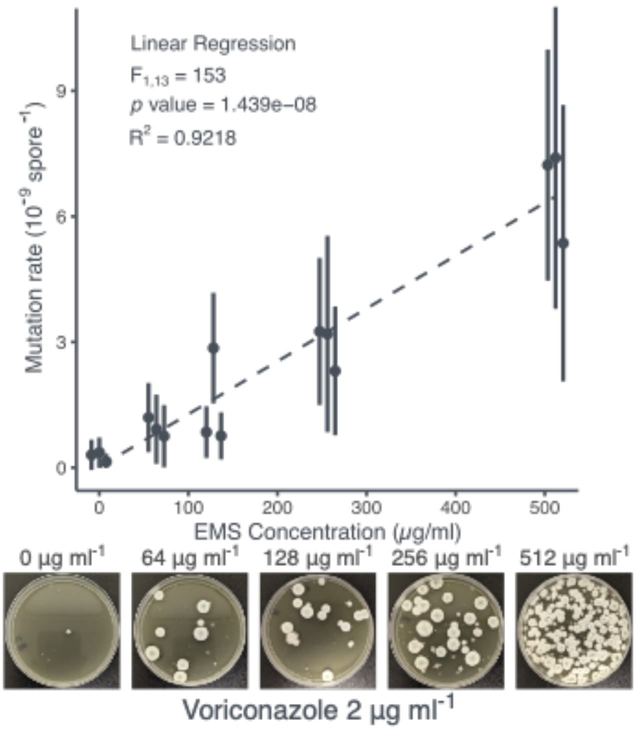

Figure S3 the effect of ethyl methanesulfonate on mutation rate. Mutation rates for resistance to voriconazole for the MFIG001 reference strain. Each point shows the calculated mutation rate from a single independent fluctuation test using 12 replicate cultures. Error bars show 95% confidence intervals. Dashed line shows linear regression fit. Images of example selective plates shown below plot. Source data are provided as a Source Data file.

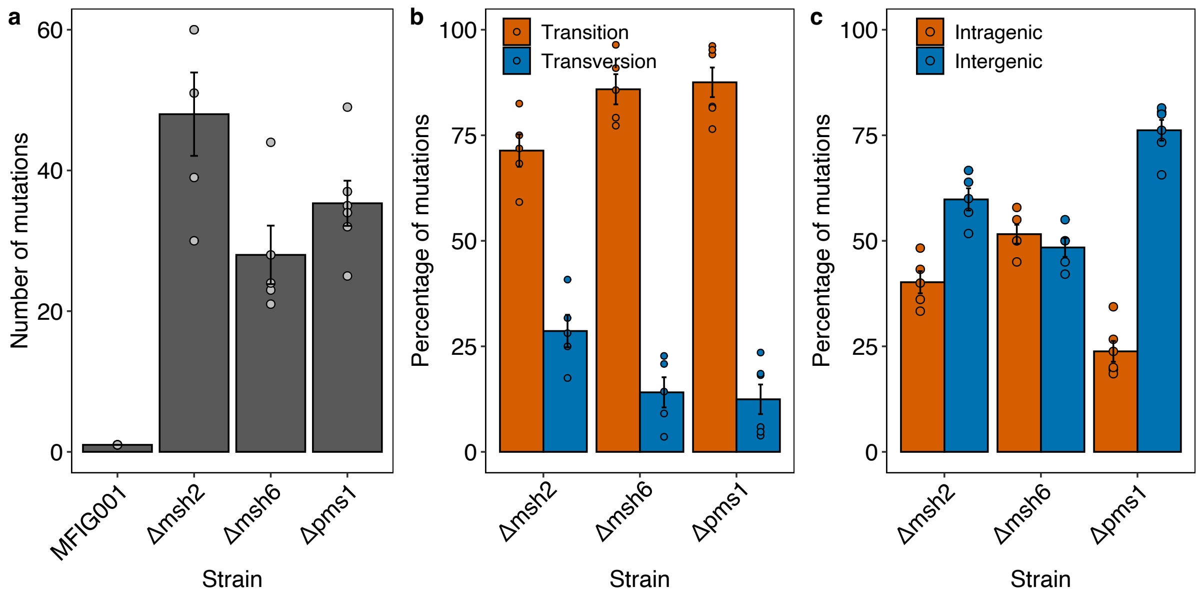

Figure S4 Mutations present in spontaneous voriconazole resistant mutants. a The number of mutations present in spontaneous voriconazole resistant mutants generated in wildtype MFIG001 (N = 6), *Δmsh2* (N = 5), *Δmsh6* (N = 5) and *Δpms1* (N = 6) backgrounds. **b** The percentage transition and transversion SNVs in each MMR deletion background. **c** The percentage intragenic and intergenic mutations in each MMR deletion background. Across all plots bars represent means, error bars standard error of the mean and points show individual spontaneous voriconazole resistant mutants. Source data are provided as a Source Data file.

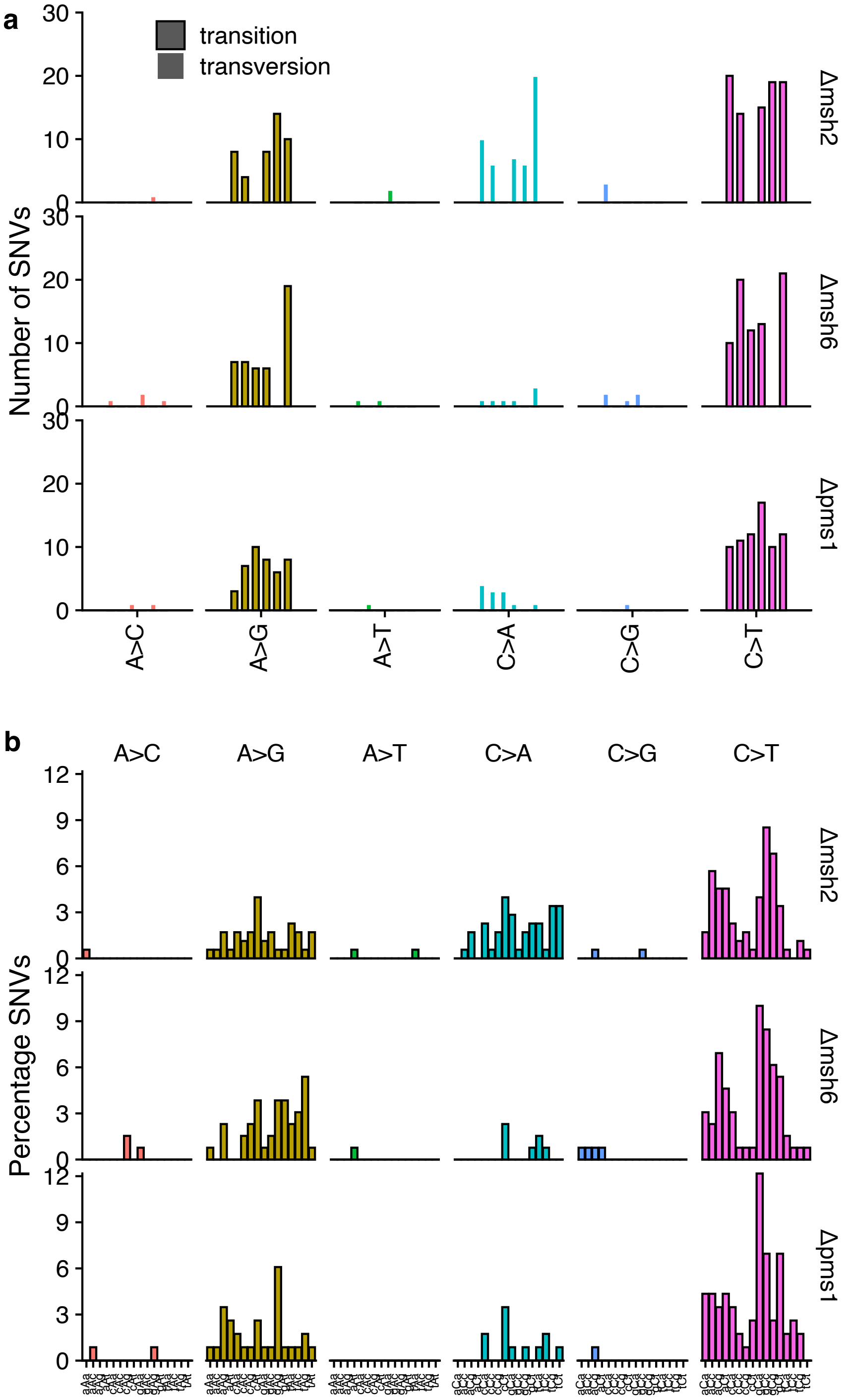

Figure S5 Identity of transitions/transversions, and trinucleotide signature. a The number of specific substitutions in each MMR deletion background. Each bar represents individual spontaneous voriconazole resistant mutants. Bars outlined in black show transitions. **b** The percentage of SNVs across all independent spontaneous voriconazole resistant mutants for each trinucleotide background. Source data are provided as a Source Data file.

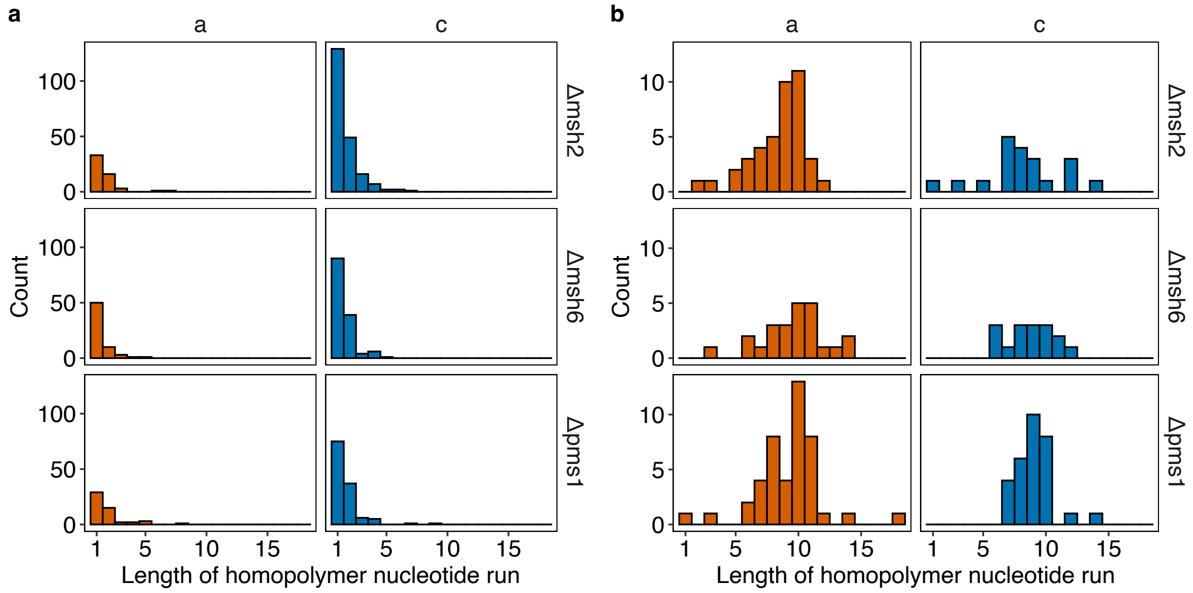

Figure S6 The occurrence of SNV and indels in homopolymer runs. a Histogram of the lengths of homopolymer runs where SNVs occur. **b** Histogram of the lengths of homopolymer runs where single base pair indels occur. Plots faceted by MMR deletion background and nucleotide in the run (a = adenine/thymine, c = cytosine/guanine). Source data are provided as a Source Data file.

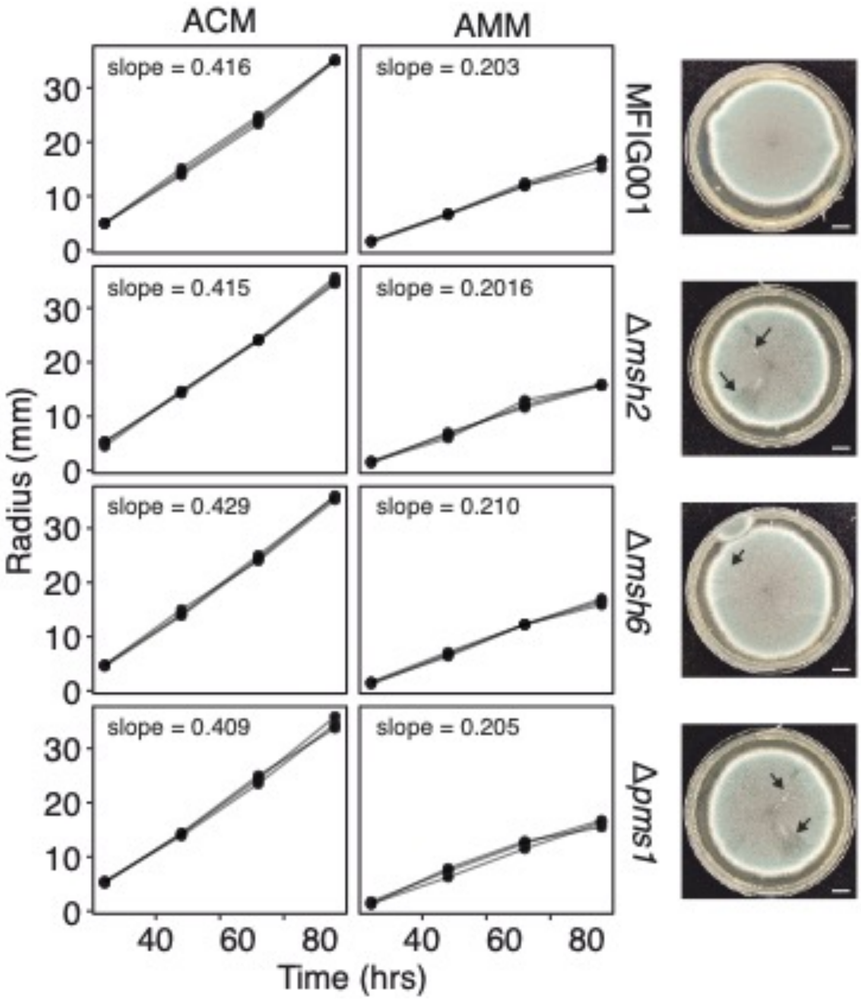

Figure S7 Radial growth rates of MMR deficient mutants. Radial growth rate of MFIG001 parental strain and *Δmsh2*, *Δmsh6* and *Δpms1* mutants in aspergillus complete media (ACM) and aspergillus minimal media (AMM), N = 3. Images of growth after 96 hours, arrows highlight presence of sectoring. Scale bar represents 10 mm. Source data are provided as a Source Data file.

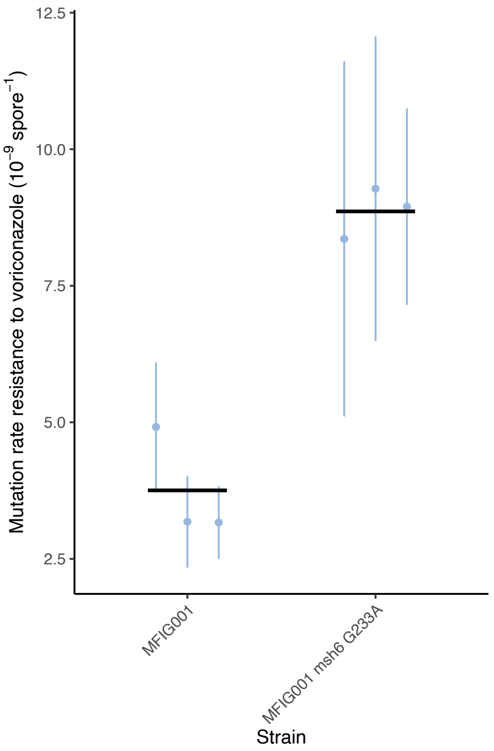

Figure S8. *msh6* G233A elevates mutation rates to voriconazole resistance in a neutral background. Each point shows the calculated mutation rate from a single independent fluctuation test using 12 replicate cultures. Each of the three points for the MFIG001 *msh6*-G223A is a separate independent transformant. Error bars show 95% confidence intervals. Introducing the *msh6*- G233A allele into the clade B isolate MFIG001 increased its mutation rate for resistance to voriconazole 3-fold (Two sample ML-test, T = -3.2727, P < 0.01). Source data are provided as a Source Data file.

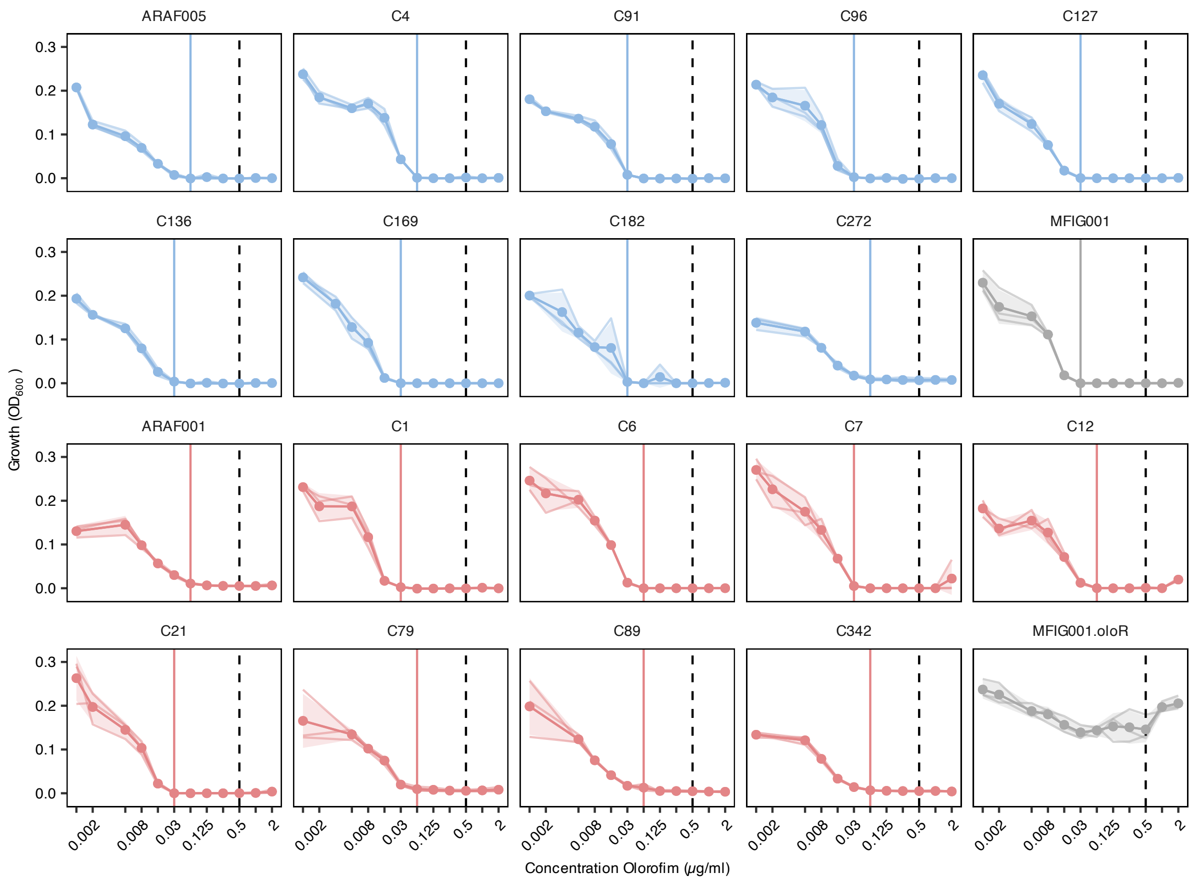

Figure S9 Olorofim MIC curves of natural isolates. Olorofim MIC curves of 18 natural isolates, MFIG001 and MFIG001 pyrG G119C, resistant to olorofim (MFIG001.oloR). Bold lines show means of three replicates, each of which are shown separately. Shaded area shows +/- standard deviation, N = 3. Dashed vertical line shows concentration used to select for resistant mutants, vertical solid line shows MIC. Coloured by clade, red clade A, blue clade B, grey MFIG001 isolates. Source data are provided as a Source Data file.

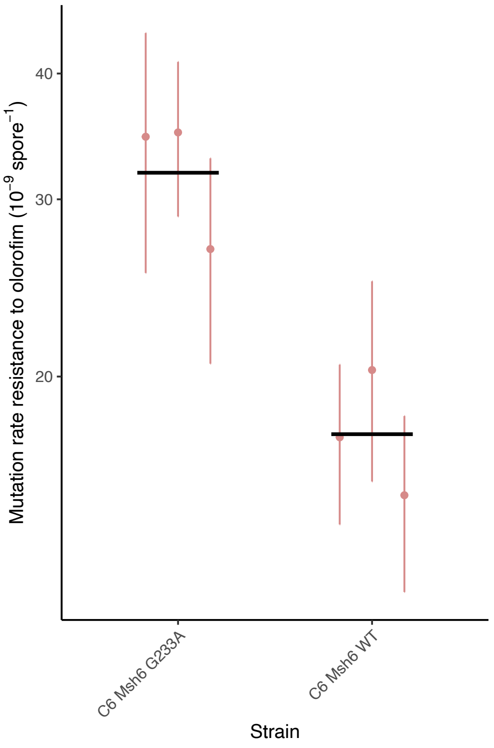

Figure S10. Reverting *msh6*-G233A to WT in a clade A isolate reduces mutation rate. Each point shows the calculated mutation rate from a single independent fluctuation test using 12 replicate cultures. Each of the three points for the C6 *msh6* WT is a separate independent transformant. Error bars show 95% confidence intervals. Reverting the msh6 allele from G233A to WT in the clade A isolate halved its mutation rate for resistance to olorofim (Two sample ML-test, $T = -3.7781$, $P < 0.001$). Source data are provided as a Source Data file.
