# Supplementary material for: Elevated mutation rates in multi-azole resistant Aspergillus fumigatus drive rapid evolution of antifungal resistance
Source: Nat Commun. 2024 Dec 16;15:10654. doi: 10.1038/s41467-024-54568-5 (PMC11649685; doi:10.1038/s41467-024-54568-5)
Supplement: Supplementary file 2 — Description of Additional Supplementary Files [file 41467_2024_54568_MOESM2_ESM.pdf]

Description of Additional Supplementary Files

File Name: Supplementary Data 1
Description: Metadata of 218 UK whole genome sequenced isolated from Rhodes et al. 2024 including annotation of variants in mismatch repair genes pms1, msh2, msh3, msh6, and mlh1.

File Name: Supplementary Data 2
Description: Variants observed in whole genome sequenced spontaneous voriconazole resistant mutants generated from MFIG001, Δmsh2, Δmsh6, and Δpms1.

File Name: Supplementary Data 3
Description: Primers, homology directed repair templates and CrispR guide RNAs used in this study.
