# Supplementary material for: Elevated mutation rates in multi-azole resistant Aspergillus fumigatus drive rapid evolution of antifungal resistance
Source: Nat Commun. 2024 Dec 16;15:10654. doi: 10.1038/s41467-024-54568-5 (PMC11649685; doi:10.1038/s41467-024-54568-5)
Supplement: Supplementary file 6 — Reporting Summary [file 41467_2024_54568_MOESM6_ESM.pdf]

nature portfolio

Corresponding author(s): Michael Bottery
Michael Bromley

Last updated by author(s): Nov 4, 2024

Reporting Summary

Nature Portfolio wishes to improve the reproducibility of the work that we publish. This form provides structure for consistency and transparency in reporting. For further information on Nature Portfolio policies, see our Editorial Policies and the Editorial Policy Checklist.

Statistics

For all statistical analyses, confirm that the following items are present in the figure legend, table legend, main text, or Methods section.

n/a	Confirmed	
☐	☒	The exact sample size (*n*) for each experimental group/condition, given as a discrete number and unit of measurement
☐	☒	A statement on whether measurements were taken from distinct samples or whether the same sample was measured repeatedly
☐	☒	The statistical test(s) used AND whether they are one- or two-sided<br>*Only common tests should be described solely by name; describe more complex techniques in the Methods section.*
☒	☐	A description of all covariates tested
☐	☒	A description of any assumptions or corrections, such as tests of normality and adjustment for multiple comparisons
☐	☒	A full description of the statistical parameters including central tendency (e.g. means) or other basic estimates (e.g. regression coefficient) AND variation (e.g. standard deviation) or associated estimates of uncertainty (e.g. confidence intervals)
☐	☒	For null hypothesis testing, the test statistic (e.g. *F*, *t*, *r*) with confidence intervals, effect sizes, degrees of freedom and *P* value noted<br>*Give P values as exact values whenever suitable.*
☒	☐	For Bayesian analysis, information on the choice of priors and Markov chain Monte Carlo settings
☒	☐	For hierarchical and complex designs, identification of the appropriate level for tests and full reporting of outcomes
☒	☐	Estimates of effect sizes (e.g. Cohen's *d*, Pearson's *r*), indicating how they were calculated

Our web collection on statistics for biologists contains articles on many of the points above.

Software and code

Policy information about availability of computer code

Data collection	Genome sequences were collected on the Illumina Novaseq 6000 platform.
Data analysis	Orthologous sequences of mismatch repair genes msh2 (AFUB_039320, AFUA_3G09850), msh3 (AFUB_090020, AFUA_7G04480), msh6 (AFUB_065410, AFUA_4G08300), pms1 (AFUB_029050, AFUA_2G13410) and mlh1 (AFUB_059270, AFUA_5G11700) were extracted from the previously published pan-genome by identifying the representative pan-genes using BLASTP v2.12.0. Extracted sequences underwent multiple-sequence alignment using MUSCLE v3.8.1551 and variants were identified using SNP-sites v2.5.1 and confirmed by visualization in JalView v2.11.2.6. Whole-genomic single nucleotide polymorphism (SNP) phylogeny of UK isolates was produced as described in Rhodes et al. Phylogenetic trees with overlaying metadata were generated using iTOL v6.5.4. Per-site FST, the measure of population differentiation due to genetic structure, was calculated for Clade A vs Clade B. It was assumed that isolates with the msh6 variant will mostly be found in Clade A, using VCFtools v0.1.13.<br><br>For analysis of global isolates publicly available raw reads (Source data file) were mapped to Af293 (GCF_000002655.1) using Burrows-Wheeler Aligner v0.7.17. Text pileup outputs were generated for each sequence using SAMtools v1.6 mpileup with option –I to exclude insertions and deletions. BCFtools v1.6 call was used to call SNPs with options -c to use the original calling method and --ploidy 1 for haploid data. Consensus genome sequences in fasta format were extracted from vcf files using seqtk v1.2 (available at https://github.com/lh3/seqtk [github.com]) with bases with a phred quality score below 40 counted as missing data. MEGA version X was used to create a neighbour-joining tree using the Tamura-Nei model with 100 bootstraps for all whole genome sequences. The phylogeny was visualized using iTOL.<br><br>Sequence logos were created using WebLogo.<br><br>For sequence data produced in this study read quality was assessed using FastQC, sequence adapters were trimmed using Trimmomatic

v0.39. De novo assembled genomes were produced for each of the parental voriconazole sensitive strains using SPAdes v3.15.4. Low complexity regions were masked using RepeatMasker v.4.1.2-p1, gene annotations were generated through a BLASTn v2.9.0 search using A1163 reference genes obtained from FungiDB (release 65), against the masked genomes. Trimmed reads from the spontaneous voriconazole resistant mutants were aligned to the appropriate parental de novo assembly using the Burrows–Wheeler Aligner MEM v0.7.17-r1188 and converted to sorted BAM format using SAMtools v.1.3.1. Variant calling was conducted using GATK HaplotypeCaller 4.1.8.0 and low confidence calls were filtered using VariantFiltration (DP < 10, RMSMappingQuality < 40.0, QualByDepth < 2.0, FisherStrand > 60.0, ABHom < 0.9). Binary presence/absence of variants in each strain were created using SAMtools "bcftools" and variants were manually confirmed using igv-reports.

All statistics were conducted in R v4.1.1, packages used for statistical analyses were: flan (v0.9) and nlme (v3.1-166).

For manuscripts utilizing custom algorithms or software that are central to the research but not yet described in published literature, software must be made available to editors and reviewers. We strongly encourage code deposition in a community repository (e.g. GitHub). See the Nature Portfolio guidelines for submitting code & software for further information.

Data

Policy information about availability of data

All manuscripts must include a data availability statement. This statement should provide the following information, where applicable:

- Accession codes, unique identifiers, or web links for publicly available datasets
- A description of any restrictions on data availability
- For clinical datasets or third party data, please ensure that the statement adheres to our policy

The sequencing data generated in this study have been deposited in the European Nucleotide Archive database under accession no. PRJEB81974 [https://www.ebi.ac.uk/ena/browser/view/PRJEB81974]. Whole genome sequence data of UK isolates was conducted as parot of Rhodes et al.20, with reads deposited under accession no. PRJEB27135 [https://www.ebi.ac.uk/ena/browser/view/PRJEB27135]. All other data generated in this study are provided in the Supplementary Information/Source Data file.

Research involving human participants, their data, or biological material

Policy information about studies with human participants or human data. See also policy information about sex, gender (identity/presentation), and sexual orientation and race, ethnicity and racism.

Reporting on sex and gender	N/A
Reporting on race, ethnicity, or other socially relevant groupings	N/A
Population characteristics	N/A
Recruitment	N/A
Ethics oversight	N/A

Note that full information on the approval of the study protocol must also be provided in the manuscript.

Field-specific reporting

Please select the one below that is the best fit for your research. If you are not sure, read the appropriate sections before making your selection.

☒ Life sciences ☐ Behavioural & social sciences ☐ Ecological, evolutionary & environmental sciences

For a reference copy of the document with all sections, see nature.com/documents/nr-reporting-summary-flat.pdf

Life sciences study design

All studies must disclose on these points even when the disclosure is negative.

Sample size	At least 3 biological replicates each of which with 12 independent cultures were used to calculate mutation rates. This provided sufficient statistical power to fit the numbers of spontaneous resistant mutants across the 12 independent cultures to Luria–Delbruck distributions to accurate mutation rate estimations.<br><br>No power calculations were conducted.<br><br>All other experiments used sample sizes that reflect those used in prior studies of this nature.
Data exclusions	No data was excluded from the analysis.
Replication	All experiments included biological replication. When testing phenotypes of genetically modified organisms independent transformants

Replication	produced in seperate transformations were tested to minimize the effect of target modifications (KO strains two independent transformants, msh6 allelic replacement 3 independent transformants).
Randomization	No randomization was conducted, all experiments included relevant positive and negative controls.
Blinding	No blinding was conducted, all experimentation was conducted. Data collection and analysis was performed by the same person who was not blinded to the conditions of the experiments. Biological experiments were impossible to conduct under the conditions of blinding.

Reporting for specific materials, systems and methods

We require information from authors about some types of materials, experimental systems and methods used in many studies. Here, indicate whether each material, system or method listed is relevant to your study. If you are not sure if a list item applies to your research, read the appropriate section before selecting a response.

Materials & experimental systems

n/a	Involved in the study
☒	☐ Antibodies
☒	☐ Eukaryotic cell lines
☒	☐ Palaeontology and archaeology
☒	☐ Animals and other organisms
☒	☐ Clinical data
☒	☐ Dual use research of concern
☒	☐ Plants

Methods

n/a	Involved in the study
☒	☐ ChIP-seq
☒	☐ Flow cytometry
☒	☐ MRI-based neuroimaging

Plants

Seed stocks	N/A
Novel plant genotypes	N/A
Authentication	N/A
